# Prevalence and factors associated with inter-arm systolic and diastolic blood pressure differences: results from the baseline Fasa Adult’s Cohort Study (FACS)

**DOI:** 10.1186/s12889-024-17857-8

**Published:** 2024-02-01

**Authors:** Ali Mouseli, Mehdi Sharafi, Zahra Amiri, Azizallah Dehghan, Elham Haghjoo, Mohammad Ali Mohsenpour, Mohammad Hassan Eftekhari, Hossein Fatemian, Omid Keshavarzian

**Affiliations:** 1 Social Determinants in Health Promotion Research Center, Hormozgan Health Institute, Hormozgan University of Medical Sciences, Bandar Abbas, Iran; 2 Noncommunicable Disease Research Center, Fasa University of Medical Sciences, Fasa, Iran; 3 Social Determinants in Health Promotion Research Center, Health Institute, Hormozgan University of Medical Sciences, Bandar Abbas, Iran; 4 Department of Persian Medicine, Fasa University of Medical Sciences, Fasa, Iran; 5 Department of Clinical Nutrition, School of Nutrition and Food Sciences, Shiraz University of Medical Sciences, Shiraz, Iran; 6 Student Research Committee, Shiraz University of Medical Sciences, Shiraz, Iran; 7 School of Medicine, Shiraz University of Medical Sciences, Shiraz, Iran

**Keywords:** Blood pressure, Blood pressure determination, Fasa adult’s cohort study, FACS, Inter-arm blood pressure difference

## Abstract

**Background:**

One of the modifiable risk factors for cardiovascular diseases is the inter-arm blood pressure difference (IAD), which can be easily measured. This study aimed to determine the prevalence and factors related to the Iranian population’s inter-arm differences in systolic and diastolic blood pressure.

**Method:**

This cross-sectional study was conducted on the baseline data of participants who had Iranian nationality, were at least 1 year of residence in the area, aged within the age range of 35–70 years, and willed to participate from the Fasa Persian Adult Cohort Study (FACS). IAD for systolic and diastolic blood pressure was measured and categorized into two groups of difference < 10 and ≥ 10 mmHg. Logistic regression was used to model the association between independent variables and IAD.

**Results:**

The prevalence of systolic and diastolic IAD ≥ 10 mmHg was 16.34% and 10.2%, respectively, among 10,124 participants. According to the multivariable logistic regression models, age (adjusted odds ratio (aOR): 1.019 [95% CI: 1.013, 1.025]), body mass index (BMI) (aOR: 1.112 [95% CI: 1.016, 1.229]), having type 2 diabetes (aOR _Yes/No_: 1.172 [95% CI: 1.015, 1.368]), having chronic headaches (aOR _Yes/No_: 1.182 [95% CI: 1.024, 1.365]), and pulse rate (aOR: 1.019 [95% CI: 1.014, 1.024]) significantly increased the odds of systolic IAD ≥ 10 mmHg. Additionally, high socio-economic status decreased the odds of systolic IAD ≥ 10 mmHg (aOR _High/Low_: 0.854 [95% CI: 0.744, 0.979]). For diastolic IAD, age (aOR: 1.112 [95% CI: 1.015, 1.210]) and pulse rate (aOR: 1.021 [95% CI: 1.015, 1.027]) significantly increased the odds of diastolic IAD ≥ 10 mmHg. Moreover, high socioeconomic status decreased the odds of diastolic IAD ≥ 10 mmHg (aOR _High/Low_: 0.820 [95% CI: 0.698, 0.963]).

**Conclusion:**

The noticeable prevalence of systolic and diastolic IAD in general population exhibits health implications due to its’ association with the risk of cardiovascular events. Sociodemographic and medical history assessments have potentials to be incorporated in IAD risk stratification and preventing programs.

## Introduction

Cardiovascular disease is an important cause of death globally [1]. Among the modifiable risk factors for cardiovascular diseases, high blood pressure is highly amendable and can be controlled by lifestyle changes or medical treatments. According to an investigation of 182 countries, hypertension prevalence was varied from 13 to 41% due to the characteristics of countries [2]. Moreover, 10.7 and 7.8 million premature death occurred related to elevated systolic and diastolic blood pressure, respectively [3]. Therefore, much attention is paid to the blood pressure of at-risk individuals [4].

According to the latest high blood pressure management guidelines, blood pressure should be checked in both arms [5, 6]. Despite these recommendations, bilateral measurement of blood pressure is often not performed in routine clinical practice [7, 8]. In this respect, an investigation showed that 30% of hypertensive patients were falsely known as normotensive when blood pressure was measured in one arm [9]. Additionally, while blood pressure measures are nearly equal for both the arms, the difference is frequently observed in various healthy or unhealthy populations [10], which is known as inter-arm difference (IAD). The proposed upper limit of normal for IAD is ≥ 10 mmHg [11], with higher values that can be associated with cardiovascular risks [12].

Previous studies reported that systolic and diastolic IAD ≥ 10 mmHg prevalence ranged 1.4–38% and 7-14.5%, respectively [13]. A meta-analysis of 60 studies showed that the systolic IAD ≥ 10 mmHg prevalence is found at 3.6% for general adult population, 11.2% in hypertensive individuals, and 7.4% in patients with diabetes [14]. Regarding the diastolic IAD, another meta-analysis reported a prevalence of 7% for diastolic IAD ≥ 10 mmHg among a pooled sample of 12,956 participants [15].

Although, in clinical practice, the acceptable IAD threshold is 10 mmHg [16], any difference greater than 5 mmHg is proportionally associated with cardiovascular disease and mortality [11]. According to the Inter-arm Blood Pressure Difference - Individual Participant Data (INTERPRESS-IPD), analysis of 53,827 participants showed that systolic IAD was associated with all-cause and cardiovascular mortality with hazard ratios (HRs) of 1.05 (95% CI: 1.02, 1.08) and 1.06 (95% CI: 1.02, 1.11), respectively, per 5 mmHg systolic IAD. Moreover, the adjusted systolic IAD per 5 mmHg was associated with cardiovascular events in people without preexisting disease (HR: 1.04 [95% CI: 1.00, 1.08]), Framingham (HR: 1.04 [95% CI: 1.01, 1.08]), or QRISK cardiovascular disease risk algorithm version 2 (QRISK2) (HR: 1.12 [95% CI: 1.06, 1.18]) cardiovascular risk scores [11]. In addition, in recently published evidence from our center, the risk of cardiovascular diseases significantly associated with systolic IAD ≥ 15 (OR: 1.412 [95% CI: 1.099, 1.814]) and diastolic IAD ≥ 10 (OR: 1.518 [95% CI: 1.238, 1.862]) [12].

The associated factors with the presence of IAD have been controversial in previous studies duo to diversities in studied population, demographic characteristics, ethnicity, and IAD measurement method. Nonetheless, factors like age, sex, socioeconomic status, occupational and educational status, high-risk behavers such as smoking and alcohol consumption, anthropometric indices, comorbid conditions like type 2 diabetes, hypertension and anxiety, etc. However, the majority of such studies have been conducted in developed countries [17–19]. Additionally, there are few pieces of evidence about the prevalence and factors associated with IAD in Iran. Therefore, this study aimed to determine the prevalence and factors related to systolic and diastolic IAD in an Iranian population.

## Methods

### Study population

This cross-sectional study was conducted on the baseline data of the Fasa Persian Adult Cohort Study (FACS). The FACS was started in 2014 in a southern area of Fars province, Iran, in the Sheshdeh and Qara-Balagh regions with a minimum anticipated sample size of 10,000. The inclusion criteria comprised being Iranian nationality, at least 1 year of residence in the area, and within the age range of 35–70 years, as well as willing to participate in the study and able to communicate verbally. People who refused to participate in the study after three phone calls were excluded from the study. The main purpose of the Persian Fasa cohort study is to investigate the risk factors for cardiovascular diseases. So far, there have done 5 follow-up periods annually for the outcome of interest occurrences, including the diagnosis of chronic diseases such as heart and brain strokes; type 2 diabetes, hypertension, cancers, and cardiovascular and vascular failure. In this study, the baseline data of the FACS were investigated for the factors related to the inter-arm differences in systolic and diastolic blood pressure. This data was initiated to collect in 2014 and was completed at the end of 2015, after including 10,124 participants out of 10,622 eligible population, which yielded a participation rate of 95.3% [20].

### Variables

#### Dependent (outcome) variable

Inter-arm blood pressure was measured. Firstly, according to the standard procedure of FACS, the systolic and diastolic blood pressures were measured for both arms twice at 10 min intervals in standard status with the calibrated device. To achieve blood pressure differences, the mean of measurements for systolic and diastolic blood pressure for both arms was calculated, and the differences between the means were recorded. Based on the previous study, differences were categorized into two groups of difference < 10 and ≥ 10 mmHg [21].

#### Independent variables

Demographic characteristics of participants included age, gender, and socioeconomic status. The socio-economic status was obtained using the asset index, since it has fewer fluctuations compared to the measurements based on the income of individuals or the amount of expenses of individuals. This asset index was developed using the state of property of each participant, comprising ownership or renting of residential home, the area of the house, number of rooms, bathroom and toilet, having a telephone, mobile phone, washing and dishwasher machines, microwave, freezer, television, refrigerator, vacuum cleaner, personal computer or laptop, motorcycle, and car or truck, and internet access at home, as well as the approximated prices of these items. Then, principal component analysis (PCA) was conducted to quantitatively generate asset index as a measure of socio-economic status of participants. The calculated asset index was further categorized into five quintiles, in which the first quintile represented the low socio-economic status, while the fifth quintile represented the high socio-economic status.

Clinical data of participants included waist circumference, smoking and opium use, self-declared past medical history (i.e., diabetes, depression, fatty liver disease, and chronic headache)– which confirmed after evaluating the provided documents by the participant, heart rate, and sleep duration at night, evening, and night. All data were extracted from the FACS database.

Moreover, after being in a 10-12-hour fasting state, a 25-ml blood sample was collected from each participant, using Vacutainers (manufacture). A small amount of blood sample was used for biochemical measurements, including fasting blood glucose (FBG) level, and concentrations of serum high-density lipoprotein cholesterol (HDL-c) and triglycerides (TG), using [manufacture] kits and an autoanalyzer (manufacture). The remaining sample was centrifuged, fractioned into 2D cryotubes (manufacture), transferred to the Cohort Reference Laboratory located at the Fasa University of Medical Sciences, and stored in a -70° C freezer for further laboratory assessments [20].

Body Mass Index (BMI), and the triglyceride-glucose (TyG) index, and TG/HDL-c index were calculated using the standard formula:

$$ \text{B}\text{M}\text{I}\hspace{0.17em}=\hspace{0.17em}\text{w}\text{e}\text{i}\text{g}\text{h}\text{t} \left(\text{k}\text{g}\right)/ \text{h}\text{e}\text{i}\text{g}\text{h}\text{t}^{2} \left(\text{m}\right),$$

$$ \text{T}\text{y}\text{G} \,\text{i}\text{n}\text{d}\text{e}\text{x}\hspace{0.17em}=\hspace{0.17em}\text{l}\text{n} \left[\text{T}\text{G} \right(\text{m}\text{g}/\text{d}\text{L}) \times \text{F}\text{B}\text{G} (\text{m}\text{g}/\text{d}\text{L})/2],$$

$$ \text{T}\text{G}/\text{H}\text{D}\text{L}-\text{c} \text{r}\text{a}\text{t}\text{i}\text{o}\hspace{0.17em}=\hspace{0.17em}\text{T}\text{G} (\text{m}\text{g}/\text{d}\text{L})/\text{H}\text{D}\text{L}-\text{c} (\text{m}\text{g}/\text{d}\text{L}).$$

### Statistical analysis

To describe the study variables, stratified by both of the systolic and diastolic IAD groups, frequency (percentage) and mean (standard deviation (SD)) were used for qualitative and quantitative variables, respectively. Independent t-tests and chi-square tests were used to compare IAD groups for various independent variables. Variables with a significance level less than 0.25 in the univariable test were entered into the multivariable model to control the effect of confounders. Logistic regression was used to model the association between dependent variables and covariates (independent variables). An adjusted odds ratio (aOR) with a 95% confidence interval (CI) was used to report the correlation effect size. The analyses were conducted using Stata software version 14. A P-value less than 0.05 was considered significant.

## Results

Of the cohort population, 10,124 entered the study. The prevalence of systolic and diatonic IAD ≥ 10 mmHg was 16.34% [95%CI: 15.63–17.08] and 10.26% [95%CI: 9.67–10.87]. The mean age and BMI of participants with systolic and diastolic IAD ≥ 10 mmHg were 50.34 ± 9.87 years and 26.09 ± 4.86 kg/m^2^, and 49.80 ± 9.85 years and 25.94 ± 5.01 kg/m^2^, respectively. Among participants with systolic and diastolic IAD ≥ 10 mmHg, 263 (15.89%) and 880 (84.70%) had diabetes, respectively. Other characteristics of the participants are shown in Table 1. Based on the univariable analysis, sex, age, socioeconomic status, BMI, waist circumference, TyG quantile index, opium use, diabetes, total daily sleep hours, afternoon sleep hours, pulse rate, fatty liver disease, and chronic headaches, were associated with presence of systolic IAD ≥ 10 mmHg. (*p* < 0.05). Additionally, for diastolic IAD ≥ 10 mmHg, the variables sex, age, socioeconomic status, BMI, waist circumference, opium use, diabetes, pulse rate, and chronic headaches were statistically significant (*p* < 0.05) (Table 1).

**Table 1:** Patient characteristics of the study population at baseline by Inter-arm systolic and diastolic IAD

Variable	Systolic IAD			Diastolic IAD		
	< 10 mmHg	≥ 10 mmHg	P	< 10 mmHg	≥ 10 mmHg	P
**Sex**, ***n (%)***						
Male	3,899 (46.04)	673 (40.66)	< 0.001	4,152 (45.70)	420 (40.42)	0.001
Female	4,570 (53.96)	982 (59.34)		4,933 (54.30)	619 (59.58)	
Age, *y (M ± SD)*	48.30 ± 9.48	50.34 ± 9.87	< 0.001	48.50 ± 9.53	49.80 ± 9.85	< 0.001
**Socioeconomic status**, ***n (%)***						
Low	2,762 (32.63)	613 (37.06)	0.001	2,984 (32.86)	391 (37.67)	0.003
Middle	2,830 (33.43)	546 (33.01)		3,036 (33.43)	340 (32.76)	
High	2,873 (33.94)	495 (29.93)		3,061 (33.71)	307 (29.58)	
BMI, *kg/m*^*2*^*(M ± SD)*	25.56 ± 4.85	26.09 ± 4.86	< 0.001	25.61 ± 4.83	25.94 ± 5.01	0.036
Waist circumference, *cm(M ± SD)*	92.89 ± 11.73	94.47 ± 12.04	< 0.001	93.06 ± 11.76	93.88 ± 12.05	0.035
**Quantile TyG index**, ***n (%)***						
Q1	2,136 (25.23)	394 (23.82)	0.014	2,273 (25.03)	257 (24.76)	0.654
Q2	2,118 (25.02)	410 (24.79)		2,266 (24.95)	262 (25.24)	
Q3	2,145 (25.34)	386 (23.34)		2,285 (25.16)	246 (23.70)	
Q4	2,067 (24.42)	464 (28.05)		2,258 (24.86)	273 (26.30)	
**Quantile TG/HLD-c ratio**, ***n (%)***						
Q1	2,112 (24.98)	413 (25.02)	0.325	2,263 (24.96)	262 (25.24)	0.886
Q2	2,108 (24.93)	418 (25.32)		2,263 (24.96)	263 (25.34)	
Q3	2,141 (25.33)	386(23.38)		(25.12) 2,278	249 (23.99)	
Q4	2,093 (24.76)	434(26.29)		2,263 (24.96)	264 (25.43)	
**Smoking**, ***n (%)***						
No	6,133 (72.42)	1,236 (74.68)	0.058	6,593 (72.57)	776 (74.69)	0.146
Yes	2,336 (27.58)	419 (25.32)		2,492 (27.43)	263 (25.31)	
**Opium use**, ***n (%)***						
No	6,666 (78.71)	1,347 (81.39)	0.014	7,160 (78.81)	853 (82.10)	0.013
Yes	1,803 (21.29)	308 (18.61)		1,925 (21.19)	186 (17.90)	
**Type 2 diabetes**, ***n (%)***						
No	7,484 (88.37)	1,392 (84.11)	< 0.001	7,996 (88.01)	880 (84.70)	0.002
Yes	985 (11.63)	263 (15.89)		1,089 (11.99)	159 (15.30)	
**Depression**, ***n (%)***						
No	7,892 (93.19)	1,553 (93.84)	0.334	8,479 (93.33)	966 (92.97)	0.664
Yes	577 (6.81)	102 (6.16)		606 (6.67)	73 (7.03)	
**Fatty liver disease**, ***n (%)***						
No	7,618 (89.95)	1,460 (88.22)	0.034	8,158 (89.80)	920 (88.55)	0.210
Yes	851 (10.05)	195 (11.78)		927 (10.20)	119 (11.45)	
**Chronic headaches**, ***n (%)***						
No	7,194 (84.95)	1,361 (82.24)	0.005	7,702 (84.78)	853 (82.10)	0.024
Yes	1,275 (15.05)	294 (17.76)		1,383 (15.22)	186 (17.90)	
Pulse Rate, *BPM (M ± SD)*	73.67 ± 10.65	76.19 ± 10.78	< 0.001	73.81 ± 10.69	76.43 ± 10.61	< 0.001
Total sleep duration, *h (M ± SD)*	7.77 ± 1.86	7.66 ± 1.83	0.035	7.76 ± 1.86	7.72 ± 1.85	0.521
Night sleep duration, *h (M ± SD)*	6.92 ± 1.60	6.87 ± 1.59	0.226	6.92 ± 1.60	6.87 ± 1.59	0.315
Afternoon sleep duration, *h (M ± SD)*	0.84 ± 0.90	0.79 ± 0.85	0.027	0.83 ± 0.89	0.85 ± 0.88	0.642

*Abbreviations* M ± SD, mean  ±  standard deviation; BMI, body mass index; IAD, inter-arm blood pressure difference

The multivariable logistic regression showed that for every year increase in age, the odds of systolic IAD ≥ 10 mmHg significantly increased by 1.019 [95% CI: 1.013, 1.025]. In addition, for each one unit increase in BMI and pulse rate, the odds of systolic IAD ≥ 10 mmHg significantly increased by 1.112 [95% CI: 1.016, 1.229] and 1.019 [95% CI: 1.014 to 1.024], respectively. Moreover, having type 2 diabetes (aOR _Yes/No_: 1.172 [95% CI: 1.015, 1.368]) and chronic headaches (aOR _Yes/No_: 1.182 [95% CI: 1.024, 1.365]) significantly increased the odds of systolic IAD ≥ 10 mmHg. Contrary, people with high socioeconomic status had around 0.15 less odds for having systolic IAD ≥ 10 mmHg compared to those with low socioeconomic status (aOR _High/Low_: 0.854 [95% CI: 0.744, 0.979]) (Table 2).

**Table 2:** Final multivariable logistic regression models for assessment of associations between covariates and Inter-arm systolic and diastolic IAD

Variable	Systolic IAD mmHg ^1^			Diastolic IAD mmHg ^2^		
	aOR	95% CI	P	aOR	95% CI	P
Age	1.019	1.013, 1.025	< 0.001	1.112	1.015, 1.210	< 0.001
**Socioeconomic status**						
Low	Ref.	-	-	Ref.	-	-
Middle	0.908	0.797, 1.034	0.146	0.886	0.758, 1.034	0.127
High	0.854	0.744, 0.979	0.024	0.820	0.698, 0.963	0.016
BMI	1.112	1.016, 1.229	0.003	-	-	-
**Type 2 diabetes**						
No	Ref.	-	-	-	-	-
Yes	1.172	1.015, 1.368	0.043			
**Chronic headaches**						
No	Ref.	-	-	-	-	-
Yes	1.182	1.024, 1.365	0.022			
Pulse rate	1.019	1.014, 1.024	< 0.001	1.021	1.015, 1.027	< 0.001

*Abbreviations* aOR, adjusted odds ratio; CI, confidence interval; BMI, body mass index; IAD, inter-arm blood pressure difference

^1^Adjusted for sex, waist circumference, quantile TyG index, smoking, opium use, fatty liver, total sleep duration, night sleep duration, and afternoon sleep duration

^2^Adjusted for sex, waist circumference, BMI, smoking, opium use, diabetes, fatty liver, and chronic headaches

For diastolic IAD ≥ 10 mmHg, the logistic regression model showed that with each year increase in age, the odds of having diastolic IAD ≥ 10 significantly increased by 1.112 [95% CI: 1.015, 1.210]. In addition, with each one unit increase in pulse rate, the odds of diastolic IAD ≥ 10 significantly increased by 1.021 [95% CI: 1.015, 1.027]. Furthermore, high socioeconomic status statistically decreased the odds of diastolic IAD (aOR _High/Low_: 0.820 [95% CI: 0.698, 0.963]) (Table 2).

## Discussion

The present study indicated that in 16.3% and 10.2% of the FACS population had IAD ≥ 10 mmHg, respectively. This observation represented a higher prevalence in comparison to other studies on general population. For example, Kimurra et al. [22] showed a prevalence of 9.1% in the Japanese population. Additionally, Johansson et al. [23], found a prevalence of 10.1% in the Finnish population. The Framingham study resulted that 9.4% of the participants had systolic IAD ≥ 10 mmHg [24]. Noticeably, two meta-analyses found a pooled prevalence of 3.6% and 7% for systolic and diastolic IAD ≥ 10 mmHg in general adult population [14, 15], which were lower than our observation. The discrepancies in the observed prevalence may be explained by differences in blood pressure measurement methods and the population of each study. In the present study, blood pressure was obtained sequentially twice in each arm in a sitting position. Hypertension guidelines recommend repeated blood pressure measurements in a sitting position to obtain accurate blood pressure records [25, 26]. However, based on the results of a meta-analysis, the frequency of subjects with systolic and diastolic IAD ≥ 10 mmHg was significantly lower when blood pressure measurements were assessed simultaneously instead of sequentially [15]. Therefore, simultaneous measurement of blood pressure in both arms is preferred due to the prevention of overestimation of prevalence and observer bias [27]. However, simultaneous blood pressure assessment in both arms requires more equipment [28]. By and large, this issue can explain a possible overestimated IAD prevalence in the present study.

The findings of the present study stated that by every one-year increase in age, the risk of having systolic and diastolic IAD ≥ 10 mmHg was increased by 1.9% and 11.2%, respectively. In a study on 1,505 participants, Gbaguidi et al. [29], found that the probability of systolic IAD ≥ 10 mmHg significantly increased with odds of 1.1 per each 10-year increase in age. Additionally, a multi-ethnicity study of 14,618 Chinese adult participants, risk of IAD was increased by 1.71-fold in the greater than 75 years age group, compare to the 35–44 age group in the multivariable logistic regression model. Moreover, a study in a Japanese population indicated that age is the only significant predictor for systolic and diastolic IAD [22]. This finding was also found in Kranenburg et al. study [30].

In our study, the prevalence of IAD ≥ 10 mmHg was 21% in individuals with type 2 diabetes history, which is higher than in previous studies [17, 31]. Moreover, having type 2 diabetes posed 17.2% higher odds for IAD ≥ 10 mmHg. This finding was consistent with meta-analyses, showing higher IAD prevalence rates among individuals with cardiovascular comorbidity such as diabetes and hypertension [14]. In addition, Gbaguidi et al. [29], found that diabetes increased the odds of systolic IAD ≥ 10 mmHg by 1.96 folds in the multivariable model. This is while, the study done by Lane et al. [32] showed that the IAD was not associated with age, gender, ethnicity, arm circumference, blood pressure status, and the history of type 2 diabetes and cardiovascular disease. Such discrepancy might be explained by used of simultaneous measurement in Lane et al. study, since sequential measurement method can overestimate IAD prevalence to threefold, as well as different studied population– a tertiary hospital– compare to our study.

Our observations showed that for each one unit increase in BMI, the chance of having systolic IAD ≥ 10 mmHg significantly increased by 11.2%. This finding was in-line with various studies conducted by Essa et al. [13], Kimura et al. [22], Gopalakrishnan et al. [33], White et al. [34], Tokitsu et al. [35], Sun et al. [36], and Song et al. [37]. Obesity usually accompanies with hypertension in individuals and is known as an important risk factor for atherosclerosis. In this regard, some authors have suggested measure blood pressure in both arms in the presence of the IAD ≥ 10 mmHg due to an increased risk of hidden atherosclerosis [19].

Another finding of this study was that for each one unit increase in pulse rate, the chance of systolic and diastolic IAD ≥ 10 mmHg significantly increased 1.9% and 1.2%, respectively. According to Kimura et al. [22], there is a positive and significant correlation between IAD and pulse rate, which is consistent with the results of this study. Moreover, another study, conducted on 3,235 young healthy adults, heart rate above 90 beats per minute was associated with IAD [13].

Moreover, high socioeconomic status decreased the chance of both systolic and diastolic IAD ≥ 10 mmHg by nearly 15% and 18%, respectively, compared to those with low socioeconomic status. Although, evidence is scarce, other sociodemographic indices such as education level are found to be significantly associated with systolic IAD. That is, in a recent study carried out by Gbaguidi et al. [29], while there was no significant association between income and occupation with systolic IAD, the sensitivity analysis showed a significant association between the level of education and the difference in systolic blood pressure between arms. It is believed that one of the most important factors that increase the incidence of cardiovascular diseases is the inequality of education. Higher education level is associated with healthier behaviors, access to jobs with healthier working environments, and better access to health care [29].

One of our study’s limitations is that most of the study population were middle-aged individuals, and the inter-arm blood pressure differences could be different in other age groups such as teenagers and young adults. Therefore, the results of this study cannot be generalized to other age groups. In addition, defining the past medical history variables (i.e., diabetes, depression, and fatty liver disease, and chronic headache) through self-reporting, although retrospectively confirmed after evaluating the provided documents by the participant, could be potentially underestimated due to the undiagnosed cases.

## Conclusion

The systolic and diastolic IAD is not uncommon in general population; and owing to the high probability of association between IAD ≥ 10 mmHg and risk of cardiovascular events, routine measurement of the blood pressure difference between the two arms is important. Moreover, since various sociodemographic, such as age, BMI and socioeconomic status, as well as medical conditions like type 2 diabetes, might be associated with the IAD likelihood, such the easily assessed factors are recommended to be incorporated in selecting the groups of individuals who might take the advantage of measuring blood pressure in both arms during cardiovascular assessment.

## Data Availability

Data can be inquired from the corresponding author.
